# Effects of Subclinical Hypothyroidism on Maternal and Perinatal Outcomes during Pregnancy: A Single-Center Cohort Study of a Chinese Population

**DOI:** 10.1371/journal.pone.0109364

**Published:** 2014-10-29

**Authors:** Liang-Miao Chen, Wen-Jun Du, Jie Dai, Qian Zhang, Guang-Xin Si, Hong Yang, En-Ling Ye, Qing-Shou Chen, Le-Chu Yu, Chi Zhang, Xue-Mian Lu

**Affiliations:** 1 Department of Endocrinology, Third Affiliated Hospital, Wenzhou Medical University, Ruian, Zhejiang, China; 2 Chinese-American Research Institute for Diabetic Complications, Wenzhou Medical University, Wenzhou, China; 3 Ruian Center of the Chinese-American Research Institute for Diabetic Complications, Third Affiliated Hospital, Wenzhou Medical University, Wenzhou, China; Central South University, China

## Abstract

**Objective:**

Adverse maternal outcomes and perinatal complications are closely associated with overt maternal hypothyroidism, but whether these complications occur in women with subclinical hypothyroidism (SCH) during pregnancy remains controversial. The aim of this study was to evaluate the effects of SCH on maternal and perinatal outcomes during pregnancy.

**Methods:**

A prospective study of data from 8012 pregnant women (371 women with SCH, 7641 euthyroid women) was performed. Maternal serum samples were collected in different trimesters to examine thyroid hormone concentrations. SCH was defined as a thyroid stimulating hormone concentration exceeding the trimester-specific reference value with a normal free thyroxine concentration. The occurrence of maternal outcomes, including gestational hypertension (GH), gestational diabetes mellitus, placenta previa, placental abruption, prelabor rupture of membranes (PROM), and premature delivery; and perinatal outcomes, including intrauterine growth restriction (IUGR), fetal distress, low birth weight (LBW; live birth weight ≤2500 g), stillbirth, and malformation, was recorded. Logistic regression with adjustment for confounding demographic and medical factors was used to determine the risks of adverse outcomes in patients with SCH.

**Results:**

Compared with euthyroid status, SCH was associated with higher rates of GH (1.819% *vs*. 3.504%, *P* = 0.020; *χ* ^2^ = 7.345; odds ratio (OR), 2.243; 95% confidence interval (CI), 1.251–4.024), PROM (4.973% *vs*. 8.625%, *P* = 0.002; *χ* ^2^ = 72.102; adjusted OR, 6.014; 95% CI, 3.975–9.099), IUGR (1.008% *vs*. 2.965%, <0.001; *χ* ^2^ = 13.272; adjusted OR, 3.336; 95% CI, 1.745–6.377), and LBW (1.885% *vs*. 4.582%, *P*<0.001; *χ* ^2^ = 13.558; adjusted OR, 2.919; 95% CI, 1.650–5.163).

**Conclusions:**

The results of this study indicate that pregnant women with SCH had increased risks of GH and PROM, and their fetuses and infants had increased risks of IUGR and LBW. Thus, routine maternal thyroid function testing is necessary to improve maternal and perinatal outcomes.

## Introduction

Thyroid hormones are critical for energy production, body temperature regulation, and development modulation [1], [2]. Thyroid dysfunction is the second most frequent endocrine disease among reproductive-aged women [3]. Hypothyroidism can be overt or subclinical [4]; overt hypothyroidism is characterized by an elevated serum level of thyroid stimulating hormone (TSH>10 mIU/L) and a subnormal free thyroxine (fT4) level, whereas subclinical hypothyroidism (SCH) is characterized by an enhanced TSH level, usually beyond the upper reference limit, and a normal fT4 level. Untreated hypothyroidism is closely associated with several pregnancy-related disorders. Because fetal thyroid hormones originate almost exclusively from the maternal system before 12–14 weeks of gestation, maternal thyroid disorders in early pregnancy are closely related to fetal development. Neurological deficits in infants and juveniles, including low intelligence quotient scores, cognitive delay, and psychomotor development impairment, are the main complications induced by maternal hypothyroidism during early pregnancy [5]–[7]. Thyroid hormone deficiency beyond the first trimester also cannot be ignored, although fetal thyroid hormones are functional at this time. For example, triiodothyronine activates enzymes important for neurological development in late pregnancy [8]. Other adverse maternal outcomes and perinatal complications associated with overt maternal hypothyroidism include miscarriage, preeclampsia, preterm labor, and fetal death [9]–[11]. However, hypothyroidism is not readily recognized because it usually manifests as non-specific symptoms. SCH is often missed in pregnant women, although its prevalence is about 2–3% [12]–[15]. This condition has been associated with neurodevelopmental disorders in fetuses and infants and several adverse maternal outcomes, including gestational diabetes mellitus (GDM), preeclampsia, placental abruption, and preterm delivery [3], [5], [13], [14], [16], [17]. Women who have been previously diagnosed with SCH are at increased risk of stillbirth and GDM in subsequent pregnancies [14].

However, no consensus has been reached about the need for universal thyroid function screening and the treatment of SCH during pregnancy. The American College of Obstetricians and Gynecologists and the clinical practice guidelines of the Endocrine Society recommended the examination of thyroid function only in women with symptoms of thyroid disease or previous histories of thyroid disease and other associated conditions [18], [19]. However, this screening protocol is not sufficient because pregnant women with SCH are often asymptomatic, with no history of immune disorder. In China, the recognition and treatment of thyroid disorder in pregnant women is also insufficient and few studies have examined the possible effects of SCH on maternal and perinatal outcomes in large Chinese populations.

In the present study, universal screening of thyroid function was conducted in a Chinese population. The prevalence of SCH and associated maternal and perinatal outcomes were determined, and the risks of adverse outcomes associated with SCH were assessed.

## Materials and Methods

### Ethics statements

This study was conducted at the Third Hospital Affiliated of Wenzhou Medical University, Zhejiang, China, and the study protocol was approved by the hospital’s institutional review board. Written informed consents were obtained from all enrolled subjects prior to the study. The privacy of all subjects was guaranteed.

### Study population and thyroid function screening

Between February 2009 and February 2012, 8012 pregnant women were enrolled in this prospective study. All subjects were screened and gave birth at the hospital, and had resided in the local area for at least 5 years. Women with the following conditions were excluded: overt thyroid disorder, previous or present use of thyroxin or anti-thyroid drugs, other autoimmune disease, congenital heart disease, and elevated serum transaminase or creatinine level (reference ranges: glutamic-pyruvic transaminase, 0–55 IU/L; glutamic oxalacetic transaminase, 6–60 IU/L; serum creatinine, 40–106 umol/L).

Thyroid function was tested at the first antenatal examination. Among 8012 women recruited for this study, 1124 (14.03%) participants were tested in the first trimester, 2640 (32.95%) were tested in the second trimester, and 4248 (53.02%) were tested in the third trimester. Information about the following demographic and clinical characteristics was collected through questionnaires administered during examination: demographic characteristics (e.g., age, address, occupation, educational level, income), medical history (menstrual history, childbearing history, other diseases, medication use), health behavior (smoking and exposure to husbands’ smoking, alcohol consumption), general physical parameters (body weight, blood pressure, cardiopulmonary function, edema), obstetric parameters (fundal height, abdominal girth, fetal heart sound, pelvic examination when necessary), and laboratory assessments (screening for GDM, HIV, syphilis, routine blood and urinary tests, hepatic and renal functions, blood type, electrocardiography, electronic fetal monitoring). All data were kept in a computerized database.

### Laboratory assays and diagnosis of SCH

Fasting venous blood samples were collected in the morning from all participants. Serum was isolated after centrifugation and stored at –80°C until testing. Serum TSH and fT4 concentrations were measured by electrochemiluminescence immunoassay (DX2800; Beckman, Bremen, Germany) and associated diagnostic kits. Inter- and intra-assay coefficients of variation for each hormone were <10%. The assessment of thyroid function was based on the following local trimester-specific reference values (2.5^th^–97.5^th^ percentiles) [20]: first trimester, TSH 0.09–3.47 mIU/L and fT4 6.00–12.25 ng/L; second trimester, TSH 0.20–3.81 mIU/L and fT4 4.30–9.74 ng/L; and third trimester, TSH 0.67–4.99 mIU/L and fT4 4.56–8.50 ng/L. SCH was defined as a TSH concentration exceeding the trimester-specific reference value in combination with a normal fT4 concentration. Pregnant women with normal TSH and fT4 levels were considered to be euthyroid and served as control subjects.

### Definition of maternal and fetal outcomes

All participants underwent monthly antenatal examinations during gestation and delivery until they were discharged from the hospital. Maternal and perinatal outcomes based on specific guidelines were recorded during this period.

The following maternal outcomes were diagnosed based on individual guidelines and documented. Gestational hypertension (GH) was defined as systolic pressure >140 mmHg and/or diastolic pressure >90 mmHg after 20 weeks of gestation, with no previous history of hypertension, including preeclampsia and eclampsia [21]. Preeclampsia was defined as persistent elevated blood pressure (systolic pressure ≥140 mmHg, diastolic pressure ≥90 mmHg) with proteinuria and eclampsia as the appearance of seizure or coma in a patient with GH. GDM was defined as a plasma glucose concentration ≥95 mg/dL after fasting, ≥180 mg/dL at 1 h after a 100-g oral glucose tolerance test (OGTT), and/or ≥155 mg/dL at 2 h after a 100-g OGTT, regardless of gestational age [22]. Placenta previa was defined as the partial or complete insertion of the placenta in the lower uterine segment and placental abruption as the separation of the placenta from the uterine lining before labor [23]. Prelabor rupture of membranes (PROM) was defined as the rupture of the amniotic sac and chorion membrane prior to the onset of labor [24], [25]. A delivery occurring between 28 and 37 completed weeks of gestation was considered premature [26], [27].

The following perinatal outcomes were assessed and documented. Intrauterine growth restriction (IUGR) was defined as an estimated fetal weight below the 10^th^ percentile for gestational age [28], [29]. Fetal distress was defined as fetal heart rate <120 bpm or >160 bpm, presence of meconium, signs of abnormal fetal movement, and fetal scalp pH<7.2 [30]. The documentation of fetal distress was based on the presence of fetal distress signs before or during labor and associated complications [31]. Low birth weight (LBW) was defined as a live birth weight ≤2500 g [32]. Stillbirth was diagnosed when fetal death occurred after the 20^th^ week of pregnancy [33]. Any malformation of the eyes, ears, or face; nervous, circulatory, urinary, reproductive, musculoskeletal system, or any other organ was recorded.

### Statistical analysis

All data are expressed as means ± standard deviations or numbers and percentages. Statistical analysis was performed using the SPSS 16.0 software. Student’s *t*-test was used to compare continuous variables (maternal age, gestational age at delivery, TSH and fT4 concentrations) and the chi-squared test was used to compare categorical measures (educational level, parity, mode of delivery, exposure to husbands’ smoking, all maternal and perinatal outcomes). The risks of adverse outcomes in patients with SCH were determined by logistic regression and represented as odds ratios (ORs) and 95% confidence intervals (CIs), with adjustment for various confounding factors (maternal age, educational level, parity, gestational age at delivery, mode of delivery, exposure to husbands’ smoking). Maternal SCH and several other possible risk factors, such as maternal age, parity (nulliparity *vs*. multiparity), and exposure to husbands’ smoking (yes/no), were introduced into the logistic regression model to identify factors associated with adverse outcomes. *P*<0.05 was considered to be statistically significant.

## Results

### Maternal demographic characteristics

Maternal demographic characteristics are shown in Table 1. Of the 8012 women, 7641 (95.37%) had TSH and fT4 values within the normal reference ranges in the trimester of testing and were considered to be euthyroid, whereas 371 (4.63%) had high TSH levels coupled with normal fT4 levels and were considered to have SCH. Mean maternal age, education level, parity, gestational age at delivery, and delivery modes were similar in the two populations (Table 1). No participant smoked or drank alcohol during pregnancy, but more than 40% of women in both groups (euthyroid, 43.16%; SCH, 44.74%; *P* = 0.887) were exposed to their husbands’ smoking.

**Table 1:** Maternal demographic characteristics.

	Euthyroid (*n* = 7641)	SCH (*n* = 371)	*P*
Maternal age (years)	27.12±0.05	26.33±0.24	0.243
Educational level			0.970
Primary school	607 (7.94%)	35 (9.43%)	
Middle school	4465 (58.43%)	238 (64.15%)	
College or university	2327 (30.45%)	84 (22.64%)	
Parity			0.856
Nulliparity	6241 (81.68%)	299 (80.59%)	
Multiparity	1400 (18.32%)	72 (19.41%)	
Gestational age at delivery (weeks)	38.99±0.02	39.05±0.09	0.087
Mode of delivery			0.121
Vaginal	4876 (63.81%)	246 (66.31%)	
Forceps	16 (0.21%)	2 (0.54%)	
Cesarean	2749 (35.98%)	123 (33.15%)	
Exposure to husbands’ smoking	3298 (43.16%)	166 (44.74%)	0.887

SCH, subclinical hypothyroidism.

### Thyroid function in different trimesters among women with SCH

Table 2 presents the TSH and fT4 concentrations of patients with SCH in different trimesters. The TSH concentration was significantly lower in the first trimester than in the third trimester (*P*<0.001). The fT4 concentration was higher in the first trimester than in the second and third trimesters (*P*<0.001).

**Table 2:** TSH and fT4 concentrations in women with SCH by trimester.

Trimester	TSH concentration (mIU/L)	fT4 concentration (ng/L)
First	4.38±0.14#	8.33±0.18
Second	4.73±0.16	6.64±0.15*
Third	6.62±0.13	6.68±0.55*

TSH, thyroid stimulating hormone; fT4, free thyroxine; SCH, subclinical hypothyroidism.

# *P*<0.001 *vs*. third trimester;

*P<0.001 *vs*. first trimester.

### Maternal outcomes in the euthyroid and SCH groups

Maternal outcomes in the two groups are compared in Table 3. No significant difference in the incidence of GDM, placenta previa, placental abruption, or preterm birth was observed between groups. The incidences of GH and PROM were significantly higher in women with SCH than in euthyroid women (3.504% *vs*. 1.819%, *P* = 0.020; 8.625% *vs*. 4.973%, *P* = 0.002).

**Table 3:** Maternal outcomes of euthyroid women and those with SCH.

Outcome	Euthyroid (*n* = 7641)	SCH (*n* = 371)	*P*
GH	139 (1.819%)	13 (3.504%)	0.020
GDM	268 (3.743%)	8 (2.156%)	0.112
Placenta previa	16 (0.209%)	0 (0%)	0.378
Placental abruption	13 (0.170%)	1 (0.270%)	0.654
PROM	380 (4.973%)	32 (8.625%)	0.002
Premature delivery	268 (3.507%)	13 (3.504%)	0.997

SCH, subclinical hypothyroidism; GH, gestational hypertension; GDM, gestational diabetes mellitus; PROM, prelabor rupture of membranes.

### Perinatal outcomes in the euthyroid and SCH groups

Comparisons of selected perinatal outcomes are shown in Table 4. No significant difference in the incidence of fetal distress or stillbirth was observed between the SCH and euthyroid groups. IUGR was more frequent in women with SCH than in euthyroid women (2.965% *vs*. 1.008%, *P*<0.001). More LBW infants were delivered in the SCH group than in the euthyroid group (4.582% *vs*. 1.885%, *P*<0.001). Twenty-eight fetuses and infants had obvious malformation, and this outcome was observed more often in the SCH group than in the euthyroid group (1.078% *vs*. 0.314%, *P*<0.05).

**Table 4:** Perinatal outcomes of euthyroid women and those with SCH.

Outcome	Euthyroid (*n* = 7641)	SCH (*n* = 371)	*P*
IUGR	77 (1.008%)	11 (2.965%)	<0.001
Fetal distress	288 (3.769%)	17 (4.582%)	0.424
LBW	144 (1.885%)	17 (4.582%)	<0.001
Stillbirth	21 (0.275%)	2 (0.539%)	0.289
Malformation	24 (0.314%)	4 (1.078%)	0.038

SCH, subclinical hypothyroidism; IUGR, intrauterine growth restriction; LBW, low birth weight.

### Trimester-stratified maternal and perinatal outcomes

Tables 5–7 present comparisons of maternal and perinatal outcomes between euthyroid women and those with SCH in different trimesters. No significant difference in outcomes was noted between the two groups of women who underwent thyroid function testing in the first trimester (Table 5). However, among those tested in the second trimester, women with SCH had significantly higher incidences of stillbirth (3.704% *vs*. 0.155%, *P* = 0.006) and malformation (3.704% *vs*. 0.425%, *P* = 0.028). Among those tested in the third trimester, the incidences of PROM (10.448% *vs*. 6.055%, *P* = 0.004), IUGR (4.104% *vs*. 1.080%, *P*<0.001), and LBW (5.970% *vs*. 2.111%, *P*<0.001) were significantly higher in women with SCH than in euthyroid women. The incidence of GH was also higher in women with SCH than in euthyroid women in the third trimester, although this difference was not significant (4.478% *vs*. 2.538%, *P* = 0.056).

**Table 5:** Maternal and perinatal outcomes of euthyroid women and those with SCH in the first trimester.

Outcome	Euthyroid(*n* = 1075)	SCH(*n* = 49)	*P*
GH	11 (1.023%)	0 (0%)	1
GDM	69 (6.419%)	1 (2.041%)	0.360
Placenta previa	0 (0%)	0 (0%)	1
Placental abruption	1 (0.001%)	0 (0%)	1
PROM	35 (3.256%)	2 (4.082%)	1
Premature delivery	33 (3.070%)	0 (0%)	0.213
IUGR	6 (0.558%)	0 (0%)	1
Fetal distress	29 (2.698%)	2 (4.082%)	0.895
LBW	14 (1.302%)	1 (2.041%)	0.49
Stillbirth	5 (0.465%)	0 (0%)	1
Malformation	3 (0.279%)	0 (0%)	1

SCH, subclinical hypothyroidism; GH, gestational hypertension; GDM, gestational diabetes mellitus; PROM, prelabor rupture of membranes; IUGR, intrauterine growth restriction; LBW, low birth weight.

**Table 6:** Maternal and perinatal outcomes of euthyroid women and those with SCH in the second trimester.

Outcome	Euthyroid (*n* = 2586)	SCH (*n* = 54)	*P*
GH	27 (1.044%)	1 (1.851%)	0.441
GDM	127 (4.945%)	2 (3.704%)	1
Placenta previa	3 (0.116%)	0 (0%)	1
Placental abruption	2 (0.773%)	0 (0%)	1
PROM	109 (4.215%)	2 (3.703%)	1
Premature delivery	66 (2.126%)	1 (1.852%)	0.746
IUGR	28 (1.083%)	0 (0%)	1
Fetal distress	85 (3.287%)	0 (0%)	0.335
LBW	46 (1.779%)	0 (0%)	1
Stillbirth	4 (0.155%)	2 (3.704%)	0.006
Malformation	11 (0.425%)	2 (3.704%)	0.028

SCH, subclinical hypothyroidism; GH, gestational hypertension; GDM, gestational diabetes mellitus; PROM, prelabor rupture of membranes; IUGR, intrauterine growth restriction; LBW, low birth weight.

**Table 7:** Maternal and perinatal outcomes of euthyroid women and those with SCH in the third trimester.

Outcome	Euthyroid (*n* = 3980)	SCH (*n* = 268)	*P*
GH	101 (2.538%)	12 (4.478%)	0.056
GDM	72 (1.809%)	5 (1.866%)	0.815
Placenta previa	13 (0.334%)	0 (0%)	1
Placental abruption	10 (0.257%)	1 (0.373%)	0.512
PROM	241 (6.055%)	28 (10.448%)	0.004
Premature delivery	173 (4.347%)	11 (4.104%)	0.85
IUGR	43 (1.080%)	11 (4.104%)	<0.001
Fetal distress	169 (4.246%)	15 (5.597%)	0.293
LBW	84 (2.111%)	16 (5.970%)	<0.001
Stillbirth	12 (0.301%)	0 (0%)	1
Malformation	10 (0.251%)	2 (0.746%)	0.056

SCH, subclinical hypothyroidism; GH, gestational hypertension; GDM, gestational diabetes mellitus; PROM, prelabor rupture of membranes; IUGR, intrauterine growth restriction; LBW, low birth weight.

### Estimated risks of maternal SCH in association with adverse outcomes

The results of logistic regression analysis of possible risk factors associated with adverse outcomes are displayed in Table 8. After adjusting for maternal age, parity, gestational age at delivery, and exposure to husbands’ smoking, SCH was found to increase the likelihood of several maternal adverse outcomes. The risk of GH was more than two-fold greater among mothers with SCH (*χ* ^2^ = 7.345; adjusted OR, 2.243; 95% CI, 1.251–4.024; *P* = 0.007). Pregnant women with SCH had a higher risk of developing PROM (*χ* ^2^ = 72.102; adjusted OR, 6.014; 95% CI, 3.975–9.099; *P*<0.001). Maternal SCH was also identified as a risk factor for fetal IUGR (*χ* ^2^ = 13.272; adjusted OR, 3.336; 95% CI, 1.745–6.377; *P*<0.001) after adjusting for confounding factors. Nearly three-fold more LBW infants were born to mothers with SCH compared with euthyroid women (*χ* ^2^ = 13.558; adjusted OR, 2.919; 95% CI, 1.650–5.163; *P*<0.001) after adjustment. However, the association of perinatal malformation with SCH was not significant in the adjusted analysis (*P* = 0.101).

**Table 8:** Logistic regression model to identify adverse outcomes associated with maternal SCH.

Outcome	*χ* ^2^	Adjusted OR	95% CI	*P*
GH	7.345	2.243	1.251–4.024	0.007
PROM	72.102	6.014	3.975–9.099	<0.001
IUGR	13.272	3.336	1.745–6.377	<0.001
LBW	13.558	2.919	1.650–5.163	<0.001
Malformation	2.687	NA	NA	0.101

SCH, subclinical hypothyroidism; OR, odds ratio; CI, confidence interval; GH, gestational hypertension; PROM, prelabor rupture of membranes; IUGR, intrauterine growth restriction; LBW, low birth weight; NA, not available or not applicable. ORs adjusted for maternal age, parity, gestational age at delivery, and exposure to husbands’ smoking.

## Discussion

The current study was performed to gain insight into the impacts of SCH on maternal and perinatal outcomes. In our study sample, 4.63% of pregnant women were diagnosed with SCH. Pregnant women with SCH had increased risks of developing GH and PROM. Fetuses and infants of women with SCH had significantly higher risks of IUGR and LBW compared with those born to euthyroid mothers.

Universal thyroid function screening before pregnancy is not currently recommended; thyroid hormone concentrations are typically measured only in women at high risk of thyroid disorders, and screening for thyroid dysfunction in early pregnancy is controversial [19]. Some clinicians support the testing of all pregnant women at the first maternity visit, and certainly by the 9^th^ week of gestation, whereas others examine only women at high risk. However, this targeting of high-risk women has been shown to overlook a significant proportion of affected women. Vaidya et al. [34] reported that targeted testing of women with personal and/or family histories of thyroid or other autoimmune dysfunction missed approximately one-third of pregnant women with overt hypothyroidism or SCH in a sample of 1500 patients. Another study suggested that targeted testing overlooks more than half of thyroid abnormalities [35]. Thus, a more extensive screening protocol for thyroid disorders may be required. Moreover, given the one child per family policy in China, pregnant women prefer to undergo thorough screening to identify any abnormality potentially affecting the health of their precious single child [36]. Thyroid function testing in pregnant women at the first prenatal visit is thus a reasonable approach in China.

Pregnancy has pronounced effects on thyroid physiology [37], [38]. Total concentrations of triiodothyronine and thyroxine, major hormones secreted by the thyroid, increase during pregnancy because of elevated thyroxin-binding globulin concentration. Human chorionic gonadotropin (HCG), a weak thyroid activator, is elevated during the first trimester of pregnancy and can trigger a slight decrease in the serum TSH concentration [39]. Thus, the serum TSH concentration is low in the first trimester, and then increases significantly in the second and third trimesters [37]. The fT4 level typically increases during the period of peak HCG level in the first trimester, and declines later in pregnancy [37]. Thus, the use of gestational-age–specific threshold values for thyroid hormones is essential for the accurate diagnosis of thyroid disorders, such as SCH [3], [40]. Trimester-specific reference values for thyroid function have been established for the Chinese population using women with normal singleton pregnancies [20]. The incidence of SCH among pregnant women in our study (4.63%), defined using these reference values, is similar to previously reported values (2–5%) [41].

The impacts of SCH on maternal and perinatal outcomes have not been clearly identified. Some studies [12], [42] revealed that SCH did not result in poor maternal outcomes, whereas other investigations demonstrated that SCH was associated with several obstetric complications, including GH, IUGR, placental abruption, and GDM [15]–[17], [43]. Thyroid hormones regulate cardiovascular activities and blood pressure, and long-term thyroid hormone disorder results in cardiovascular dysfunction [44]–[48]. In non-pregnant adults, SCH was also associated with the higher incidence and recurrence of congestive heart failure compared with euthyroid adults [48]. In the current study, the incidence of GH was higher in women with SCH than in euthyroid women in the third trimester, although this difference was not significant. More importantly, when data from all three trimesters were analyzed together, the incidence of GH was significantly higher in women with SCH than in euthyroid women after adjusting for confounding factors, such as maternal age, parity, gestational age at delivery, and exposure to husbands’ smoking. This association has been supported by molecular research documenting decreased nitric oxide secretion and impaired endothelium-related vasodilation in patients with SCH [49], which could be reversed by thyroxine replacement.

A higher risk of GDM associated with SCH has also been reported. In a large population-based study involving 24,883 women, the risk of GDM increased markedly with elevated TSH concentration during pregnancy [15]. Although another study by Cleary-Goldman and colleagues [12] documented a higher risk of GDM associated with overt hypothyroidism (adjusted OR, 1.7; 95% CI, 1.02–2.84), no such association was observed in patients with SCH. In the present study, we also failed to demonstrate that SCH affected GDM development. This difference may be due to the use of different cutoff values to define SCH (TSH concentration >4.13 mIU/L with no correction for gestational age in Tudela et al. [15] *vs*. gestational-age–specific ranges of TSH concentration in the present study).

The present study documented a higher risk of PROM in pregnant women with SCH, especially in the third trimester, which did not differ according to maternal age, parity, or smoking status. Although no other study has reported that SCH is associated with an increased risk of PROM, several reports have suggested that this risk is higher in patients with overt hypothyroidism. A higher incidence of PROM was observed in patients with hypothyroidism than in healthy pregnant women (11.7% *vs*. 7.8%, *P*<0.001) in a large population-based cohort study [50]. Davis et al. [51] also found a high incidence of PROM in subsequent euthyroid pregnancies among previously overtly hypothyroid women. In our sample, the incidence of IUGR was almost four-fold greater in fetuses of mothers with SCH than in euthyroid mothers after adjustment for confounding factors and smoking status. Similar observations have been described in other reports. Ohashi et al. [39] reported IUGR in 25% of pregnancies involving maternal thyroid dysfunction, and 16.52% (19/115) of IUGR cases occurred in the SCH group. We also found that SCH was associated with a significant risk of LBW, as reported by Leung et al. [52]. This association was not very evident in the first and second trimesters, but the incidence of LBW was almost three times higher among women with SCH than among euthyroid women in the third trimester, which could also be due the higher incidence of IUGR in those women. These findings suggest that the increased rate of LBW in infants born to women with SCH is related to this thyroid disorder. Because IUGR and LBW are reported risk factors for subnormal neurobehavioral performance and intellectual development [53]–[55], possible links between IUGR and LBW in infants born to mothers with SCH and impaired psychological development have been proposed [6], [56]. Although we found that the incidence of malformation was higher in infants born to women with SCH than in those born to euthyroid women, particularly in the second trimester, this association was not significant after adjustment for confounding factors. Casey et al. [16] and Mannisto et al. [57] also suggested that maternal thyroid disorder was not associated with an increased rate of fetal malformation. Su and colleagues [58] reported higher risks of circulatory system (one case, 11.1%) and musculoskeletal (two cases, 4.7%) malformations in the fetuses of women with hypothyroidism. However, their sample was small and the higher incidence of fetal malformation was observed in women with clinical hypothyroidism (higher TSH level and lower fT4 level) and isolated hypothyroidism (normal TSH level and low fT4 level). In the present study, the stillbirth rate was higher among women with SCH than among euthyroid women in the second trimester, but no significant difference was observed in analysis of the entire sample. The association of a higher stillbirth rate with SCH in the second trimester may be the result of a combination of various adverse complications. Casey et al. [16] also found no difference in stillbirth rate according to thyroid status. Although an increased stillbirth rate in hypothyroid women with TSH levels >10 mU/L has been previously reported, SCH (characterized by much lower TSH levels) seems to have little influence on stillbirth [16].

The strengths of this investigation are related to the prospective examination of a large population-based cohort. The major finding of this study was that SCH, a relatively common disorder in pregnant women, has pronounced effects on maternal and fetal outcomes. Specifically, SCH can lead to GH and PROM in mothers, and higher incidences of IUGR and LBW in infants. However, this study has several limitations. First, it was based on data from a single center. Second, the impacts of anti-thyroid antibodies, including thyroglobulin and thyroid peroxidase antibodies were not taken into account when assessing maternal and fetal outcomes. Our results may have been affected by the presence of thyroid antibodies, which may increase the possibility of developing hypothyroidism or be directly associated with adverse obstetric outcomes, regardless of thyroid hormone status [19], [59]. Third, because the serum TSH level may be elevated in overweight and obese women, SCH may be mistakenly diagnosed in such patients [60]. Body mass index was not accounted for in this study, which may have affected the results. Finally, the interplay of SCH and adverse outcomes, or those among different outcomes, were not addressed in this study. For example, GH, especially preeclampsia, can raise the TSH level [61]. Hence, this study could not determine whether SCH caused the increased rate of GH or vice versa. Furthermore, preeclampsia is a major cause of IUGR due to reduced nutrition transportation from the placenta [62], [63]. Thus, this study could not determine whether the presence of SCH increases the risk of GH, which is further related to an increased incidence of IUGR, or whether SCH is directly related to the increased prevalence of IUGR.

Our results suggest that SCH is associated with several fetal and infant defects, as well as maternal dysfunction. Further investigation of the effects of thyroid function screening and prospective medical intervention on SCH in a randomized, placebo-controlled experiment could aid the verification of these associations with adverse obstetric outcomes.
